# A systematic review of the pathophysiology of 5-fluorouracil-induced cardiotoxicity

**DOI:** 10.1186/2050-6511-15-47

**Published:** 2014-09-04

**Authors:** Anne Polk, Kirsten Vistisen, Merete Vaage-Nilsen, Dorte L Nielsen

**Affiliations:** 1 Departments of Cardiology, Herlev Hospital, University of Copenhagen, Herlev Ringvej 75, DK-2730 Herlev, Denmark; 2 Departments of Oncology, Herlev Hospital, University of Copenhagen, Herlev Ringvej 75, DK-2730 Herlev, Denmark

**Keywords:** 5-fluorouracil, Cardiotoxicity, Pathophysiology, Systematic review

## Abstract

**Background:**

Cardiotoxicity is a serious side effect to treatment with 5-fluorouracil (5-FU), but the underlying mechanisms are not fully understood. The objective of this systematic review was to evaluate the pathophysiology of 5-FU- induced cardiotoxicity.

**Methods:**

We systematically searched PubMed for articles in English using the search terms: 5-FU OR 5-fluorouracil OR capecitabine AND cardiotoxicity. Papers evaluating the pathophysiology of this cardiotoxicity were included.

**Results:**

We identified 27 articles of 26 studies concerning the pathophysiology of 5-FU-induced cardiotoxicity. The studies demonstrated 5-FU-induced: hemorrhagic infarction, interstitial fibrosis and inflammatory reaction in the myocardium; damage of the arterial endothelium followed by platelet aggregation; increased myocardial energy metabolism and depletion of high energy phosphate compounds; increased superoxide anion levels and a reduced antioxidant capacity; vasoconstriction of arteries; changes in red blood cell (RBC) structure, function and metabolism; alterations in plasma levels of substances involved in coagulation and fibrinolysis and increased endothelin-1 levels and N-terminal-pro brain natriuretic peptide levels. Based on these findings the proposed mechanisms are: endothelial injury followed by thrombosis, increased metabolism leading to energy depletion and ischemia, oxidative stress causing cellular damage, coronary artery spasm leading to myocardial ischemia and diminished ability of RBCs to transfer oxygen resulting in myocardial ischemia.

**Conclusions:**

There is no evidence for a single mechanism responsible for 5-FU-induced cardiotoxicity, and the underlying mechanisms might be multifactorial. Further research is needed to elucidate the pathogenesis of this side effect.

## Background

5-Fluorouracil (5-FU) and capecitabine are chemotherapeutics used to treat solid cancers, including gastrointestinal cancers, breast cancer, head and neck cancer and pancreatic cancer. Capecitabine is a 5-FU pro-drug, that is converted to 5-FU inside tumour cells [1]. A severe side effect to 5-FU and capecitabine-based treatment is cardiotoxicity, which often presents as myocardial ischemia, but to a lesser extent cardiac arrhythmias, hyper- and hypotension, left ventricular dysfunction, cardiac arrest and sudden death [2-7]. The incidence of 5-FU-induced cardiotoxicity varies between 0-35% and may depend on dose, cardiac comorbidity and schedule of chemotherapy [2,3,5].

The clinical handling of 5-FU-induced cardiotoxicity is difficult as the pathophysiological mechanisms underlying this cardiotoxicity remain undefined [2,8-13]. Several mechanisms have been proposed, including vascular endothelial damage followed by coagulation, ischemia secondary to coronary artery spasm, direct toxicity on the myocardium and thrombogenicity due to altered rheological factors. The present review addresses the pathophysiology of 5-FU- and capecitabine-induced cardiotoxicity and discusses the evidence for the proposed mechanisms.

## Method

This systematic review is conducted according to the PRISMA guidelines [14] (Additional file 1).

### Search strategy

We searched PubMed (1966–2013) for publications in English using the search string: (5-FU or 5-fluorouracil or capecitabine) AND cardiotoxicity. The last search was carried out in October 2013. Additionally we hand-searched reference lists of retrieved papers.

### Study selection process

All citations retrieved were reviewed on full citation, abstracts and indexing terms (where provided in the databases) by two authors independently. They were rated as “relevant”, “possibly relevant” or “not relevant”. Full-text publications of all potentially relevant articles were reviewed for eligibility independently by the same two authors. All disagreements in rating or eligibility were resolved by discussion of the full-text articles till consensus was reached. All articles or abstracts in English exploring the pathophysiology of 5-FU or capecitabine cardiotoxicity were eligible. Case reports were excluded. Full articles were obtained, and references were checked for additional relevant articles.

### Data extraction

The studies were grouped into in vitro studies (studies on cultured cells or cell lines), ex vivo animal studies (conducted on functional organs that had been removed from the intact organism), in vivo animal studies (conducted on living organisms in their normal intact state) and human studies. One author extracted the following data from all studies where provided: the type of study (in vitro, ex vivo animal, in vivo animal or human), the experimental model used, the number of tests objects, the parameters evaluated, the methods applied and the results of the performed tests.

## Results

**Figure 1:**
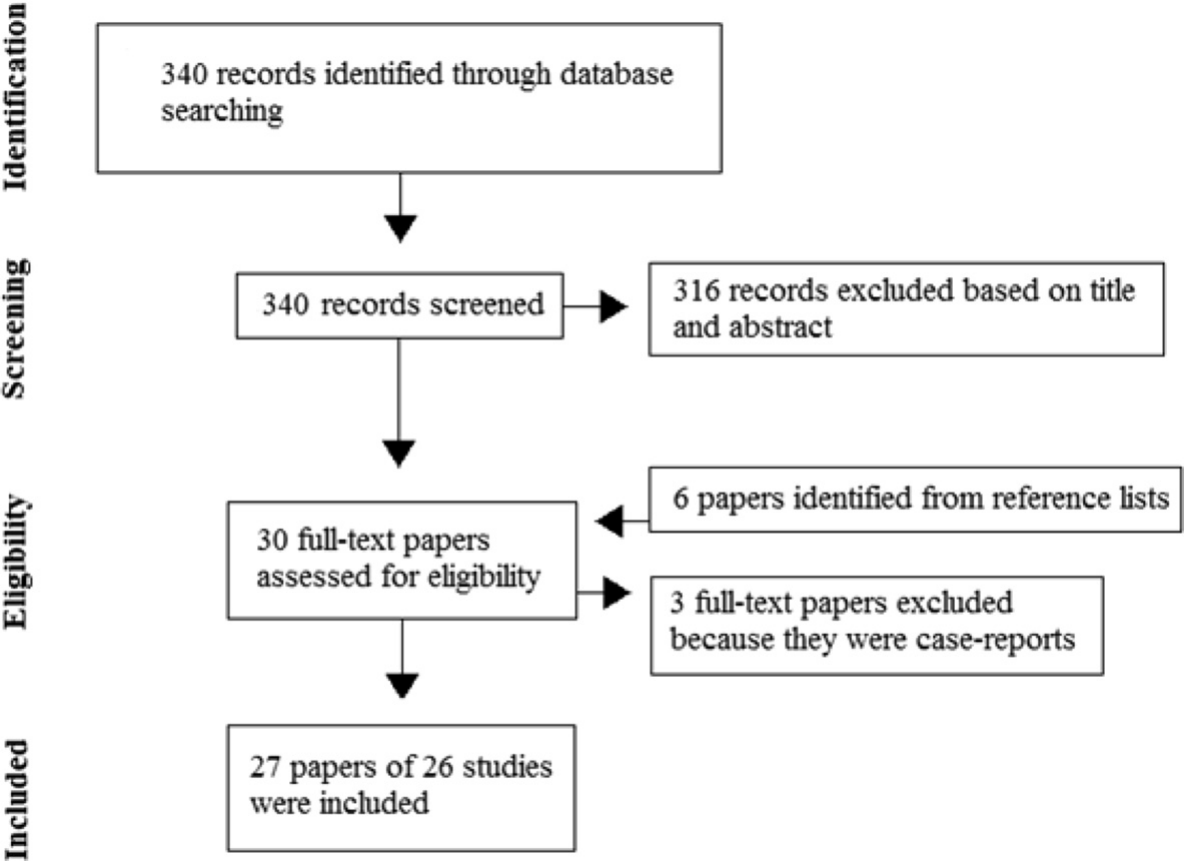
Study identification and selection process.

Twenty-seven papers of 26 studies were included (eight in vitro studies, two ex vivo animal studies, nine in vivo animal studies, six human studies and one study with results from both in vitro and human experiments) (Figure 1). All studies evaluated the pathophysiology of 5-FU-induced cardiotoxicity. We did not identify any studies evaluating the pathophysiology of capecitabine-induced cardiotoxicity. Additional file 2 shows a table with characteristics and results of the included studies. The positive, negative and conflicting findings from these studies are summarized in Table 1.

**Table 1:** Positive, negative and conflicting findings from the included studies

**Myocardium and hemodynamic function**	
Positive	Hemorrhagic infarction, interstitial fibrosis and inflammatory reaction in the myocardium [15,16]
	Induction of apoptosis and increased oxidative stress [17]
	Impaired antioxidant defense system and lipid peroxidation [18,19]
	Depletion of high energy phosphate compounds and accumulation of citrate in the myocardium [12]
	Increased oxygen consumption [20]
	Positive chronotropic effect on the sinus node [21]
Conflicting or unclarified	Increased/no change in iron levels in the myocardium [18,19]
	Positive/negative inotropic effect on the heart [20,21]
	Increased/no change in myocardial blood flow [12,20]
Negative	No changes in magnesium, potassium, calcium or copper levels in the myocardium [18,19]
	No changes in blood pressure and heart rate [12]
**Arteries**	
Positive	Endothelial damage followed by platelet aggregation [9,22-24]
	Decreased DNA synthesis, decreased total cellular protein levels and increased prostacyclin release from endothelial cells [25]
	Vasoconstriction of arteries [26-28]
**Rheological factors and RBCs**	
Positive	Changes in erythrocyte structure, function and metabolism [29-32]
Negative	Decreased and not increased blood and plasma viscosity [33]
**Substances in blood samples**	
Positive	Rise in fibrinopeptide A activation and reduction in protein C activity compared with protein C antigen [10]
	Decrease in fibrinogen levels [33]
	Decrease in coagulation factors II + VII + X and increase in lactic acid, NT-proBNP, von Willebrand factor and fibrin D-dimer levels and urine albumin-to-creatinine-ratio [8,34]
	Increased plasma levels of endothelin-1 [35]
Conflicting or unclarified	A trend towards increased levels of big endothelin [28]
Negative	No change in angiotensin II levels [27]

### Histopathological studies of the myocardium

The histopathological effects of 5-FU- were examined in two animal studies [15]. In rat hearts, multifocal interstitial hemorrhages, multifocal myofiber necrosis, inflammatory reactions including perivascular involvement, pericarditis, valvulitis and vascular changes, were found [15]. The vascular changes included dilated vessels, ruptured vascular walls, extravasation of blood and microthrombosis. In rabbits, a single high intravenous dose resulted in hemorrhagic infarction of the ventricle walls, proximal spasms of the coronary arteries and lethal outcome for all rabbits within 1 day [16]. In contrast, repeated lower doses resulted in left ventricular hypertrophy due to reticular interstitial fibrosis with edema, concentric fibrous thickening of the intima of small distal coronary arteries and disseminated foci of necrotic myocardial cells [16]. Whether the differences in histopathological effects were species specific, or due to different doses, is not clear.

### Histopathological studies of the arteries

Four studies [9,22-24] examined the histopathological effects of 5-FU on the arterial endothelium in rabbits. Scanning electron microscopy of the arteries showed extensive cytolysis, denudation of the underlying internal elastic lamina, platelet aggregation and fibrin formation [9,22-24]. Areas of contracted vessel walls with contracted endothelial cells were present [9,22-24]. Cell detachment was frequently seen and endothelial cells presented with a range of morphologic features compatible with cytolysis [9,22-24]. The endothelial damage was comparable in arteries exposed to direct injection of 5-FU and arteries exposed to 5-FU through systemic circulation [24]. The endothelial changes were most pronounced on day 3 after 5-FU injections and had diminished on day 7 and day 14 [9]. Concomitant treatment with probucol, a lipid-lowering drug with strong antioxidant properties, abrogated the effects of 5-FU on the endothelium [24], while concomitant treatment with dalteparin, a low-molecular-weight heparin, resulted in a somewhat different picture with endothelial damage on day 3, diminishing on day 7 but increasing again by day 14 [9]. Dalteparin prevented fibrin formation and to a lesser extent platelet aggregation [9].

### Studies on cultured myocardial and endothelial cells

In vitro treatment of H9c2 rat cardiomyocytes with 5-FU induced a time- and dose-dependent growth inhibition that was enhanced by levofolene [17]. Apoptosis was more frequent in 5-FU- and levofolene-treated H9c2 cells compared with colon cancer cells, and cleavage of caspase 3, an effector caspase in the apoptotic pathways, was increased in 5-FU treated H9c2 cells [17]. Moreover, superoxide anion levels increased [17]. Comparing the toxicity of 5-FU on cardiomyocytes and endothelial cells from rat hearts, Wenzel and Cosma [36] found that 5-FU induced more severe metabolic and morphological changes in endothelial cells than in cardiomyocytes. In contrast, an in vitro study of human endothelial cells and bovine endothelial cells showed no effect on cell death of 5-FU, but decreased (^3^H)thymidine incorporation, decreased total cellular protein levels and increased prostacyclin release after 5-FU incubation [25]. Taken together, these findings suggest differences in susceptibility between different cell lines to the direct toxic effects from 5-FU [17,36] and possibly even different cellular responses to the same stressor [17]. The toxic effects may not be lethal for the cells, but may reflect reversible interference with cellular function [36].

### Studies of myocardial metabolism and hemodynamic function

Studies of myocardial metabolism in guinea pigs showed that 5-FU induced a decrease in myocardial high energy phosphate levels [12,36,37] and accumulation of citrate in the myocardium [12] reflecting increased anaerobic metabolism. These changes were associated with electrocardiographic (ECG) changes suggestive of myocardial ischemia occurring around 3 hours after intravenous administration of 5-FU [12]. The depletion of high energy compounds was not associated with alterations in myocardial blood flow [12] as myocardial blood flow remained unchanged during 5-FU treatment. In contrast, Millart et al. [20] found that mean coronary flow was consistently increased in 5-FU-pre-treated rat hearts. This discrepancy can be due to different species, different experimental models or different methods to measure coronary flow. Likewise, Tamatsu et al. [37] showed that the experimental model used (open-chest or closed-chest) influenced the magnitude of depletion in high energy phosphate compounds [37].

Millart et al. [20] found no changes in oxygen uptake and cardiac contractility during perfusion of the isolated perfused rat heart with 1 mg/L 5-FU for 80 minutes. However, when rats where pre-treated with 5-FU (50 mg/kg intraperitoneally for 5 days) before killing and excision of the hearts, oxygen consumption and mean coronary flow were significantly higher compared with controls [20]. Also, a negative inotropic effect was seen in 5-FU pre-treated rats. In contrast, Satoh et al. [21] reported that 5-FU had a positive inotropic effect on the atria, and a positive chronotropic effect on the sinoatrial node, in an isolated sinoatrial node and atrial model. These conflicting findings may be due to the different animal models used.

### Studies of the myocardial antioxidant system

The influence of 5-FU treatment on the antioxidant system in myocardial tissue was studied by Durak et al. [18]. They found lowered activities of superoxide dismutase and glutathione peroxidase accompanied by higher catalase activity in 5-FU-treated female guinea pigs. The antioxidant potential, defined relative to malondialdehyde (MDA) levels, declined in 5-FU-treated animals compared with controls, while MDA levels increased [18]. A slight increase in intratissular MDA levels (not significant) and lower α-hydroxybutyrate dehydrogenase activity were demonstrated by Millart et al. [19].

The iron level was 20% higher in 5-FU-treated rat myocardial tissue compared with controls in one study [19], while another study of open-chest guinea pigs could not demonstrate increased iron levels in the myocardium after 5-FU infusion [18]. The two studies used comparable methods for iron content determination (flame atomic absorption spectrophotometry and atomic absorption spectroscopy), but they used different species and dosages. Magnesium, potassium, calcium and copper levels in myocardial tissue were unaffected by 5-FU treatment in both studies [18,19].

### Studies of vasoconstriction of arteries

5-FU-induced vasoconstriction has been demonstrated in three studies [26-28]. Two studies [27,28] showed that vasoconstriction of the brachial artery occurred in patients immediately after 5-FU infusion. The 5-FU-induced vasoconstriction was short-lived, reoccurred with repeated 5-FU injections and was abolished by glycerol nitrate [28]. In the study by Südhoff et al. [28] no patients had symptoms of cardiotoxicity, and in the study by Salepci et al. [27] three of 31 patients treated with 5-FU developed chest pain. ECG abnormalities were documented in five of 31 patients by Salepci et al. [27], while ECG recordings were not performed by Südhoff et al. [28].

Mosseri et al. [26] studied 5-FU-induced vasoconstriction in vitro using isolated aorta rings excised from rabbits. The prevalence of vasoconstriction correlated with the molar concentration of 5-FU and the magnitude was proportional to the concentration of 5-FU. The magnitude of vasoconstriction was similar for aorta rings, with functionally preserved endothelium and aorta rings with purposely disrupted endothelium indicating that an intact endothelium was not a prerequisite for 5-FU-induced vasoconstriction [26]. 5-FU-induced vasoconstriction was abolished by nitroglycerin, and acetylcholine-induced endothelium-dependent relaxation was unaffected by 5-FU-treatment [26], suggesting that 5-FU-induced vasoconstriction is not due to impairment of endothelial relaxation pathways. Pre-treatment with staurosporine, a protein kinase C (PK-C) inhibitor, reduced 5-FU-induced vasoconstriction, while pre-treatment with phorbol-12,13-dibutyrate, an activator of PK-C, increased the magnitude of 5-FU-induced vasoconstriction 23-fold [26]. In contrast, neomycin, an inhibitor of phosphoinositide turnover, and the cyclo-oxygenase inhibitor, indomethacin, did not alter the magnitude of 5-FU-induced vasoconstriction [26]. All of the membrane receptor blockers tested in the study [26], including the α-adrenergic receptor blocker phentolamine, the β-adrenergic receptor blocker propranolol, the H_1_ receptor inhibitor diphenhydramine, the H_2_ receptor inhibitor cimetidine and the Ca^2+^ channel blockers verapamil and diltiazem failed to alter the magnitude of 5-FU-induced vasoconstriction [26].

### Studies on blood rheology and red blood cells

In vitro studies of red blood cells (RBCs) incubated in 5-FU showed a dose-dependent, reversible transformation of RBCs into echinocytic shape [29,30], which resulted in impaired transit through small pores [29]. Also, alterations in membrane fluidity and RBC metabolism were observed [30-32]: potassium efflux increased [30], oxygen tension (*pO2*) decreased [31,32], deoxy-hemoglobin levels increased [32], intracellular ATP levels declined and the intracellular 2,3-bisphosphoglycerate (2,3-BPG) concentration rose [31,32]. Spasojevic et al. [32] measured ^31^P-nuclear magnetic resonance (^31^P-NMR) spectra of blood samples obtained from five patients treated with 5-FU and cisplatin, and found a downfield shift in ^31^P-NMR spectra in vivo. However, the number of patients was too small for statistical analysis.

Baerlocher et al. [29] demonstrated a continuous decrease in blood viscosity with increasing 5-FU concentrations at low shear rates (the rate of change of velocity at which one layer of fluid passes over an adjacent layer) and increasing blood viscosity at high shear rates. 5-FU had no effect on plasma viscosity in this in vitro study [29]. In contrast, plasma viscosity and blood viscosity at both natural and standardized hematocrit decreased in blood samples from 11 patients receiving 5-FU and cisplatin [33]. These inconsistent findings may have resulted from differences in exposure to 5-FU between in vitro and human studies.

### Studies of substances in blood samples from humans

Studies of the clotting-fibrinolytic system have shown increased levels of D-dimer [8] and fibrinopeptide A [10], decreased levels of fibrinogen [33] and coagulation factors II + VII + X [8], and decreased activity of the coagulation inhibitor, protein C, in blood [10]. Additionally, von Willebrand factor, which mediates the adherence and aggregation of platelets to the subendothelium, increased during 5-FU therapy [8]. However, none of the abnormalities reported in these studies was confined to patients experiencing cardiotoxicity [8,10,33]. Jensen et al. [8,34] reported that coagulation factors II + VII + X decreased during infusion, while levels of lactic acid, plasma N-terminal pro brain natriuretic peptide (NT-proBNP), von Willebrand factor, fibrin D-dimer, and the urine albumin-to-creatinine-ratio, increased. These changes were transient and only NT-proBNP levels were higher in patients experiencing cardiotoxicity [34]. A trend towards increased levels of big endothelin, a precursor to endothelin-1, was demonstrated in one study, but a large inter-individual variation was found [28]. Thyss et al. [35] reported higher plasma levels of endothelin-1 in 5-FU-treated patients compared with cancer patients receiving non-5-FU-based chemotherapy. Also, patients experiencing cardiotoxicity during 5-FU treatment had higher endothelin-1 levels compared with patients without cardiotoxicity [35]. Angiotensin II levels remained unchanged during 5-FU treatment [27]. Thus, several substances in blood were affected by 5-FU treatment, but only NT-proBNP and endothelin-1 were associated with cardiotoxicity.

## Discussion

The experimental studies and human studies included in this review showed that 5-FU induced a range of effects on the heart, on the vascular endothelium and at the cellular level of RBCs, myocardial and endothelial cells. However, to what extent these effects are involved in the pathogenesis of the clinical cardiotoxicity is more difficult to resolve. In the following, we discuss the findings and their possible role in the pathogenesis of 5-FU-induced clinical cardiotoxicity.

### Histopathological changes

Animal studies showed that 5-FU induced pathological changes in the myocardium as well as on the endothelium, in arterial vessel walls. Although the endothelial studies did not involve the coronary arteries, there was evidence of systemic endothelial injury [24]. In the myocardium, the damages seemed to depend on the dose of 5-FU administered, as high doses led to more pronounced injuries [16].

It is not clear to what extent the histopathological features demonstrated in animal studies can be found in patients experiencing clinical signs of cardiotoxicity, as no biopsy studies have been performed in patients experiencing cardiotoxicity. Human myocardial biopsy samples are hard to obtain, and it is doubtful whether they will provide new and meaningful information as most cancer patients are exposed to several drugs. Therefore, the use of experimental models is necessary to obtain better insight into the mechanisms of 5-FU-cardiotoxicity. One approach is to use cellular models of isolated cardiac myocytes, which have been extensively used to study the cardiotoxic effects of anthracyclines [38-46]. Likewise, Lamberti et al. [17] used rat H9c2 cardiomyocytes to demonstrate that 5-FU toxicity in cardiomyocytes was largely due to induction of apoptosis, as opposed to cytotoxicity in colon cancer cells, which was more likely due to necrosis or autophagy. Whether this finding reflects a different mechanism of cardiotoxicity compared with the antineoplastic effect in tumor cells, or merely different cellular responses to the same stressor, is not clear.

### 5-FU induced endothelial injury and thrombus formation

Platelet aggregation and fibrin formation on sites of endothelial injury in scanning electron microscopy studies of rabbit vascular endothelium suggested that the pathophysiological mechanism of 5-FU-induced cardiotoxicity involved a thrombogenic effect of 5-FU, secondary to endothelial injury [9,22-24]. However, the absence of vascular occlusions in many patients undergoing coronary angiography for 5-FU-induced chest pain does not support thrombosis as a main mechanism [47-63]. On the other hand, a state of ongoing intravascular coagulation was evident from studies of the clotting-fibrinolytic system. A pro-coagulant state is common in cancer patients and is triggered by tumor-produced pro-coagulant factors and tumor-cell-derived cytokines [64]. Several studies have shown that fibrin and fibrinogen degradation products are elevated in patients with colorectal cancer [65-68]. Also, von Willebrand factor is increased in cancer patients and has been correlated to advanced tumor state [69-72]. In the study by Jensen et al. [8] 47 of 106 patients had baseline plasma levels of von Willebrand factor above the reference interval before 5-FU therapy, but 97 of 106 patients had von Willebrand factor levels above the reference interval during 5-FU therapy. Hence, it is likely that both the underlying cancer disease and exposure to 5-FU contribute to the observed alterations in plasma levels of substances involved in coagulation and fibrinolysis. However, it is unlikely that these alterations play an important role in the pathogenesis of 5-FU-induced cardiotoxicity, as they were not confined to patients experiencing cardiotoxicity.

### The role of myocardial metabolism in 5-FU induced cardiotoxicity

Animal studies of myocardial metabolism demonstrated depletion of high energy phosphate compounds, citrate accumulation and increased oxygen consumption in the heart after pre-treatment with 5-FU [12,20,37]. Depletion in high energy phosphate compounds can result from increased oxygen consumption leading to insufficient oxygen supply, increased anaerobic metabolism, or to metabolic derangements produced by 5-FU. The stable and increased myocardial blood flow observed in two studies suggests that insufficient blood and oxygen supply is not a contributing factor. Instead, Suzuki et al. [73] reported that the respiratory control rate of myocardial mitochondria was significantly lower in rabbits treated with 5-FU compared with controls. Therefore, Millart et al. [20] proposed that the increase in anaerobic metabolism and the increase in oxygen uptake could be due to reduced aerobic efficiency resulting from mitochondrial uncoupling. Uncoupling of the mitochondrial respiratory chain results in increased basal oxygen consumption and decreased ATP-production. This theory should be further studied.

### The theory of oxidative stress

The pathogenesis of 5-FU induced cardiotoxicity may involve oxidative stress, as increased levels of superoxide anion were demonstrated in H9c2 cells after 5-FU treatment [17]. Reactive oxygen species (ROS), like superoxide anions, are under normal physiological conditions cleared by antioxidant defense systems, such as sodium oxide dismutase (SOD) and glutathione peroxidase (GSH-Px). Superoxide anion is dismutated to hydrogen peroxide (H_2_O_2_) in a process catalyzed by SOD, and H_2_O_2_ is then eliminated by catalase or GSH-Px [74]. The activities of SOD and GSH-Px were lowered in 5-FU treated guinea pigs [18] demonstrating a reduced antioxidant capacity. If not eliminated by cellular antioxidant systems, superoxide anions can generate the highly reactive and toxic hydroxyl radicals (-OH) through the Haber–Weiss reaction, which is catalyzed by iron [74,75]. Increased ROS levels inside cells lead to oxidation of macromolecules, including lipids, nucleic acids, and proteins, thereby disturbing cellular functions [75]. MDA is a frequently used marker of lipid peroxidation [76], and MDA levels were elevated in guinea pig hearts after 5-FU-treatment [18], and slightly elevated (but not significantly) in isolated rat hearts after 5-FU-treatment [19]. These findings indicate that some degree of oxidative stress and cellular damage takes place in animal hearts during 5-FU-treatment. Likewise, Kinhult et al. [24] suggested that 5-FU-induced damage to the arterial endothelium may be due to generation of free radicals, resulting in lipid peroxidation. Their demonstration of a protective effect of probucol on arterial endothelium in rabbits treated with 5-FU supports this statement. Probucol increases SOD and GSH-Px activities in animals, thereby improving antioxidant potential [77-79].

The role of iron and other redox active metals in formation of ROS and promotion of myocardial oxidative stress during 5-FU treatment was investigated in two studies, with conflicting results [18,19]. Increased iron levels were demonstrated in the isolated perfused rat heart by Millart et al. [19], but no changes in iron levels were found in guinea pigs by Durak et al. [18]. Iron catalyzes the formation of hydroxyl radicals, promoting oxidative stress. If iron and oxidative stress plays a role in 5-FU-induced cardiotoxicity then iron-chelators could be a possible treatment option. Taken together, the role of oxidative stress in the pathogenesis of 5-FU cardiotoxicity is not well-established, and the source of ROS formation remains undefined. In vitro studies of free radical formation and animal studies investigating the role of iron-chelators may confirm or disprove this hypothesis.

### The theory of vasospasm

The theory of vasospasm leading to myocardial ischemia has been proposed, because coronary angiography largely failed to show stenoses in patients with acute 5-FU-induced cardiotoxicity [47-63]. Moreover, coronary artery vasospasm has been visualized during coronary angiography in a few cases [80-82], and peripherally, vasoconstriction of the brachial artery appears immediately after 5-FU-injection [27,28]. It is anticipated that vasoconstriction measured peripherally after 5-FU-injection occurs in the coronary arteries as well. However, invasive methods such as cardiac catheterization and coronary angiography during infusion are necessary to prove vasospasm in the coronary arteries. While vasoconstriction is observed immediately after 5-FU injection, clinical cardiotoxicity often presents at the end of infusion, or hours to days later [2]. Moreover, cardiotoxicity may occur after several series of 5-FU or capecitabine. Hence, it remains to be elucidated in which circumstances 5-FU-induced vasoconstriction leads to clinical signs of cardiotoxicity.

In the search for the mechanism that leads to 5-FU-induced vasoconstriction, Mosseri et al. [26], exposed rabbit aorta rings to a range of substances that are involved in regulation of vascular tonus. The authors found preserved acetylcholine-induced relaxation of the vascular wall, and that glyceryl nitrate prevented 5-FU-induced vasoconstrictions [26]. Acetylcholine is an endothelium-dependent vasodilator that induces vasodilation through the NO-cGMP pathway [83]. Intact endothelial cells are a prerequisite for acetylcholine-induced vasodilation, and in the absence of endothelial cells acetylcholine leads to vasoconstriction [83]. As both acetylcholine-induced vascular relaxation and vascular relaxation by glyceryl nitrate were intact during 5-FU infusion, it seems unlikely that 5-FU causes functional vasoconstriction through impaired vasodilatory response. In contrast, Mosseri et al. [26] showed that PK-C might be a mediator of 5-FU-induced vasoconstriction. PK-C requires Ca^2+^ and phospholipid for its activation [84]. Diacylglycerol (DAG) considerably increases the affinity of PK-C for Ca^2+^, and thereby fully activates PK-C without a net increase in the Ca^2+^ concentration [84]. DAG is formed from phosphatidylinositol-4,5-bisphosphate (PIP_2)_ cleavage, but neomycin, a competitive inhibitor of the phosphodiesterase that cleaves PIP_2_, did not alter the 5-FU-induced vasoconstriction [26]. That denotes that PK-C might not be activated through cleavage of PIP_2_ and DAG formation, but rather through an unknown mechanism, or directly from 5-FU. Furthermore, there was no evidence for any modulation of 5-FU-induced vasoconstriction by membrane receptor blockers or activators of the cyclooxygenase pathway [26]. Noteworthy is that no effect of the Ca^2+^-antagonists, verapamil and diltiazem, which are often used to treat vasospasm, were seen [26].

The high plasma levels of endothelin-1 observed by Thyss et al. [35] in 5-FU treated patients, and especially in patients experiencing 5-FU-induced cardiotoxicity, may support the hypothesis of 5-FU-induced vasoconstriction. Endothelin-1 is a potent vasoconstrictor produced by endothelial cells, cardiomyocytes and cardiac fibroblasts, but it is also produced in several noncardiac tissues such as the lungs [85,86]. Endothelin-1 is known to have a regulatory role in coronary vascular resistance and myocardial capillary blood flow in coronary artery diseases [86-88]. Hypoxia, ischemia or shear stress are stimuli that induce the synthesis and secretion of endothelin-1 in vascular endothelial cells [85]. Endothelin-1 is synthesized from the precursor peptide big endothelin [85]. A trend towards increased big endothelin levels in the plasma of 5-FU treated patients was found by Salepci et al. [27], but this trend was not confined to patients who developed vasoconstrictions. As endothelin-1 and some big endothelin-1 are secreted mainly towards the adjacent smooth-muscle layer of blood vessel wall, only smaller amounts of the peptides reach the lumen of the vessel and contribute to the plasma levels [85]. Hence, it is possible that the raised endothelin-1 in plasma may come from cellular sources other than the endothelial cells. To further elucidate the role of endothelins in 5-FU-induced cardiotoxicity, the cellular source of endothelin-1 and the contribution of endothelin-1 to vasomotor tone during 5-FU infusion should be studied.

### 5-FU-induced changes in rheological factors and cardiotoxicity

Reversible transformation of RBCs into echinocytic shapes, increased membrane fluidity of RBCs and altered metabolism in terms of a rapid depletion of *pO*_*2*_, production of 2,3-BPG and decreased ATP levels [30-32] diminish the ability of RBCs to transfer oxygen to the heart. However, cisplatin induced almost similar changes in RBC morphology and membrane fluidity [32]. As cisplatin does not cause myocardial ischemia, which is the primary manifestation of 5-FU-induced cardiotoxicity, this finding might indicate that changes in RBC shape and membrane fluidity are not the principal cause of 5-FU-induced ischemia [32]. In contrast, significant depletion of *pO*_*2*_, increase in deoxy-hemoglobin levels, increase in 2,3-BPG levels and decrease in ATP levels were only observed for erythrocytes treated with 5-FU alone and erythrocytes treated with the combination of 5-FU and cisplatin. Hence, altered RBC metabolism might be a cause of 5-FU-induced ischemia. However, it is unknown whether the changes in RBC metabolism and morphology observed in vitro also occur in vivo. Also, the link between these changes and clinical signs of cardiotoxicity in patients is not proven. It is unlikely that changes in blood viscosity are a part of the pathogenesis of 5-FU induced cardiotoxicity, as studies on blood viscosity reported conflicting results [29,33].

### Other proposed mechanisms for 5-FU-induced cardiotoxicity

Few other mechanisms for 5-FU-induced cardiotoxicity have been proposed. Lemaire et al. [11] proposed that 5-FU cardiotoxicity was due to degradation products formed in the basic medium in which 5-FU is dissolved. The compound thought to be responsible for the cardiotoxicity is fluoroacetate, which is formed through alkaline hydrolysis of 5-FU and by catabolism of 5-FU in the liver [89]. However, the consistent nature of 5-FU and capecitabine-induced cardiotoxicity, regardless of the solutions or formulations used, makes it unlikely that 5-FU cardiotoxicity is due to degradation products formed in the solution of 5-FU in a basic medium. Thus, if fluoroacetate plays a role in 5-FU-induced cardiotoxicity it is likely because of metabolism of 5-FU to fluoroacetate in the liver.

Toxic myocarditis has been proposed by Sasson et al. [90], as they found biventricular dilation and diffusely scattered areas of cell necrosis associated with an inflammatory infiltrate, on autopsy of a case of 5-FU-induced fatal cardiogenic shock.

### Limitations

First, the selection of studies concerning the pathophysiology of 5-FU cardiotoxicity could not be based on objective criteria, but instead relied on the author’s judgments of which studies were concerned with the pathophysiology of 5-FU-induced cardiotoxicity. Still, we made a broad inclusion of studies that investigated the effects of 5-FU on any part of the cardiovascular system. Therefore, we believe that this review makes up a comprehensive and systematic synthesis of the results from the pathophysiological studies of 5-FU cardiotoxicity. However, as our literature search was restricted to English, a few studies may have been missed.

Second, most experimental studies included few animals and were only carried out once. Hence the statistical powers in these studies were low and the findings rarely confirmed in other studies, which makes those findings less consistent.

Third, extrapolation of results from in vitro and ex vivo studies to in vivo settings should be done with precaution, as isolated cells in vitro and isolated organs may not behave the same as in in vivo settings. There are also important differences between species, and more importantly, between animals and humans.

Fourth, some in vivo animal experiments used repeated administrations of 5-FU, but numerous studies have used regimens in which animals were treated with only a single and/or a very high 5-FU dose. In clinical practice, 5-FU is often administered according to the De Gramont schedule where a short bolus infusion is given followed by a 48-hour continuous infusion. Hence, differences in administration schedules and doses may also limit the extrapolation of the results from these studies to clinical settings.

Fifth, most of the findings in the human studies were not confined to patients experiencing cardiotoxicity, as only NT-proBNP and endothelin-1 were higher in patients with cardiotoxicity. Hence, for nearly all findings, their role in the pathogenesis of cardiotoxicity is still unclear.

Finally, some types of studies that can be conducted in animals are difficult to carry out in human patients. For example, biopsy samples are hard to obtain and carry a risk of bleeding and myocardial wall perforation for the patient. Such risks may outweigh the expected benefit for the patient, and therefore make such procedures unreasonable to perform.

## Conclusions

This review indicates that there is no evidence for a single mechanism responsible for 5-FU-induced cardiotoxicity, and the underlying mechanisms might be multifactorial. The proposed cardiotoxic pathways leading to myocardial and endothelial damage are not mutually exclusive, and they may each contribute to cardiovascular dysfunction resulting in the clinical picture of cardiotoxicity. Further research is needed to elucidate the pathogenesis of this side effect. Studies on cardiac and endothelial cell lines might contribute to further elucidation of the cellular response to 5-FU. A human study with continuous ECG monitoring concurrent with measurements of the brachial artery diameters could be interesting, to explore the theory of arterial vasospasm leading to myocardial ischemia. Other methods to further study the pathogenesis of 5-FU-induced cardiotoxicity could be studies of myocardial perfusion with magnetic resonance scanning or PET rubidium scanning of the heart in patients presenting with signs of cardiotoxicity.

## Competing interests

The authors declare that they have no competing interests.

## Authors’ contributions

AP participated in the design of the review, the literature search, the study selection process and the data synthesis, and drafted the manuscript. KV and MVN participated in the design of the review. DN participated in the design of the review, the literature search, the study selection process and helped with the data synthesis. All authors read and approved the final version of the manuscript.

## Pre-publication history

The pre-publication history for this paper can be accessed here:

http://www.biomedcentral.com/2050-6511/15/47/prepub

## Supplementary Material

Additional file 1 **PRISMA Checklist.** The PRISMA Checklist for this systematic review. Click here for file

Additional file 2 **Characteristics and results of the included studies.** This table shows the characteristics of the studies included in the review. Click here for file
