# Supplementary material for: A systematic review of the pathophysiology of 5-fluorouracil-induced cardiotoxicity
Source: BMC Pharmacol Toxicol. 2014 Sep 4;15:47. doi: 10.1186/2050-6511-15-47 (PMC4170068; doi:10.1186/2050-6511-15-47)
Supplement: Additional file 2 — Characteristics and results of the included studies. This table shows the characteristics of the studies included in the review. [file 2050-6511-15-47-S2.pdf]

Additional file 2: Characteristics and results of the studies

Histopathological studies of the myocardium	Type of study	Model	Parameters	Effects	Proposed mechanisms
Kumar et al. [2]	In vivo	Albino rats (50) (bolus 5-FU, 5-FU for 4 days, 5-FU for 7 days or 5-FU weekly for 3 weeks and controls)	Morphology	Foci of myofibre necroses and inflammatory reaction<br>Multifocal hemorrhage, valvulitis and pericarditis<br>Vascular changes: dilated vessels, ruptured vascular wall, extravasation and microthromboses	Thrombosis
Tsibiribi et al. [3]	In vivo	Rabbits (3x6: single high dose 5-FU, repeated dose 5-FU and controls)	Morphology<br>Assessment of apoptosis	Single high iv dose: Massive hemorrhagic infarct of the ventricle walls, proximal spasms of the coronary arteries. Died within 1 day.<br>Repeated dose: left ventricular hypertrophy due to reticular interstitial or perivascular fibrosis with edema, concentric fibrous thickening of the intima of small distal coronary arteries, multifocal necrosis and disseminated foci of necrotic cells	Coronary artery spasm<br>Thrombosis
**Histopathological studies of the arteries**	**Type of study**	**Model**	**Parameters**	**Effects**	
Kinhult et al. [6]	In vivo	Rabbits (15 given 5-FU, 15 given dalteparin, 15 given 5-FU + dalteparin and 15 given saline)	Endothelial morphology 1, 3, 7, 14 and 30 days after 5-FU injections	5-FU only: pronounced injuries peaking on day 3, massive cell lysis, denuded areas, fibrin formation and substantial platelet aggregation<br>5-FU-dalteparin and the saline-dalteparin: less pronounced endothelial damage on day 3, diminished on day 7, but increasing again by day 14	Endothelial injury<br>Thrombosis
Kinhult et al. [7]	In vivo	Rabbits (15 5-FU, 15 saline, 15 probucol + 5-FU, 15 probucol + saline, 15 low dose probucol + 5-FU)	Endothelial morphology 24 h, 3 days, 7 days, 14 days and 30 days after 5-FU injections	5-FU only: Extensive cytolysis, denudation of the internal elastic lamina, platelet aggregation and fibrin formation<br>Probucol + 5-FU/saline and saline only group: Minor damage as endothelial cell contraction, vessel-wall contraction and villus formation	Endothelial injury<br>Thrombosis
Cwikiel et al. [4]	In vivo	Rabbits (6 5-FU intraperitoneally, 6 5-FU intra-arterially and 6 controls)	Morphology of the endothelium in small arteries	Contracted vessel walls<br>Contracted and cytolytic endothelial cells<br>Cell detachment and denuded areas<br>Platelet accumulation and fibrin formation	Artery contraction<br>Endothelial injury<br>Thrombosis
Cwikiel et al. [5]	In vivo	Rabbits (15 5-FU intra-arterially and 5 controls)	Morphology of the endothelium in small arteries	Contracted vessel walls<br>Contracted and cytolytic endothelial cells<br>Cell detachment and denuded areas<br>Platelet accumulation and fibrin formation	Artery contraction<br>Endothelial injury<br>Thrombosis

NOT = the results do not support the proposed mechanism
AOP = antioxidant potential; CAT = catalase; GSH-PX = glutathione peroxidase; MDA = malondialdehyde; NS = non-significant; SOD = superoxide dismutase; LVEF = left ventricular ejection fraction.
[a]Shear rate = the rate of change of velocity at which one layer of fluid passes over an adjacent layer.

Additional file 2: Characteristics and results of the studies *continued*

Studies on cultured myocardial and endothelial cells	Type of study	Model	Parameters	Effects	Proposed mechanisms
Lamberti et al. [8]	In vitro	H9c2 rat cardiomyocytes treated with 5-FU +/- levofolene	Cell proliferation<br>Apoptosis<br>Oxidative stress (superoxide anion levels)	Time- and dose-dependent growth inhibition potentiated by levofolene<br>Apoptosis potentiated by levofolene<br>Increased cleavage of caspase 3<br>Increased superoxide anion levels potentiated by levofolene<br>N-acetyl cysteine abrogated the apoptotic and oxidative stress effects	Oxidative stress
Cwikiel et al. [38]	In vitro	Human endothelial cell line (HEC) and bovin endothelial cell line (BEC)	DNA synthesis: (^{3}H)thymidine incorporation<br>Total cellular protein content<br>Prostaglandin release	(^{3}H)thymidine incorporation decreased in HEC and BEC (HEC < BEC)<br>Total cellular protein content decreased in HEC and BEC<br>Prostaglandin release increased in HEC and BEC	-
Wenzel and Cosma [10]	In vitro	Beating myocytes, endothelial cells and fibroblasts from neonatal rat hearts	Metabolism (Quantitative metabolic inhibition test, QMIT)<br>Morphology	Higher QMIT grades (i.e. less acid production) with increasing concentration of 5-FU<br>TC_{50} values: Endothelial cells and fibroblasts 0.34 pg/ml and myocytes 3.25 pg/ml	Increased metabolism
**Studies of myocardial metabolism, function and antioxidant system**	**Type of study**	**Model**	**Parameters**	**Effects**	**Proposed mechanisms**
Matsubara et al. [11]	In vivo	Anesthetized open-chest guinea pigs (6x5)	Myocardial blood flow<br>ECG and heart rate<br>Analysis of tricarboxylic acid cycle intermediates in excised hearts	ST changes on ECG, more animals affected at higher doses<br>No effect on blood pressure and heart rate<br>No changes in myocardial blood flow<br>Decrease in ATP and creatine phosphate levels<br>Accumulation of citrate	Myocardial ischemia not due to decreased blood flow
Tamatsu et al. [12]	In vivo	Open-chest and closed-chest anesthetized male guinea pigs	High energy phosphate compounds	More marked depletion of myocardial high energy phosphate compounds in open-chest animals than in closed-chest animals<br>Higher mortality in open-chest animals than in closed-chest animals	Increased metabolism

NOT = the results do not support the proposed mechanism
AOP = antioxidant potential; CAT = catalase; GSH-PX = glutathione peroxidase; MDA = malondialdehyde; NS = non-significant; SOD = superoxide dismutase; LVEF = left ventricular ejection fraction.
[a]Shear rate = the rate of change of velocity at which one layer of fluid passes over an adjacent layer.

Additional file 2: Characteristics and results of the studies *continued*

Millart et al. [13]	Ex vivo	Isovolumic perfused rat heart	Contractility<br>Oxygen consumption<br>Lactate release<br>Creatine kinase activity	Negative inotropic effect<br>Increased mean coronary flow<br>Increased oxygen consumption in pretreated rat hearts<br>No change in the ratio of oxygen consumption to rate-pressure product	Decreased contractility of the heart<br>Increased aerobic myocardial metabolism
Millart et al. [16]	Ex vivo	Isovolumic perfused rat heart	Magnesium, potassium, calcium, copper and iron contents<br>MDA levels	20% higher iron levels in the 5-FU group<br>No differences for magnesium, potassium, calcium or copper<br>Lower alpha-hydroxybutyrate dehydrogenase leakage<br>Slightly higher MDA levels (NS)	NOT electrolyte changes
Satoh et al. [14]	In vitro	Isolated sinoatrial node and atrial preparation from mongrel puppies treated with 5-FU or ftorafur (15)	Action potential of the sinoatrial node cells<br>Atrial contraction	Positive chronotropic and inotropic effect of 5-FU and ftorafur<br>No alterations of pindolol, verapamil, aminophylline and low Ca2+ concentration on the positive responses of 5-FU and ftorafur	NOT decreased contractility of the heart.
Durak et al. [15]	In vivo	Female guinea pigs (5 receiving 5-FU for 5 days, 5 controls)	SOD, GSH-PX and CAT activities<br>MDA, Fe and Cu levels<br>Antioxidant potential (AOP)<br>Morphology	Decreased activities of myocardial SOD and GSH-PX, elevated CAT activity<br>Higher MDA levels<br>Fall in AOP<br>No changes in Fe and Cu levels<br>No histopathological alterations	Decreased antioxidant capacity/ oxidative stress<br>NOT ion changes
**Studies of vasoconstriction of arteries**	**Type of study**	**Model**	**Parameters**	**Effects**	**Proposed mechanisms**
Mosseri et al. [17]	In vitro	Aorta rings freshly isolated from rabbits treated with 5-FU (150)	Vasoconstriction/isometric tension	Concentration-dependent vasoconstriction<br>No alteration in ACh-induced endothelium-dependent relaxation<br>A protein kinase C inhibitor reduced 5-FU-induced vasoconstriction<br>An activator of protein kinase C increased 5-FU-induced vasoconstriction<br>Glycerolnitrate abolished 5-FU-induced vasoconstriction<br>No effect of an inhibitor of phosphoinositide turnover, a cyclooxygenase inhibitor and membrane receptor blockers on 5-FU-induced vasoconstriction	Arterial vasoconstriction leading to myocardial ischemia

NOT = the results do not support the proposed mechanism

AOP = antioxidant potential; CAT = catalase; GSH-PX = glutathione peroxidase; MDA = malondialdehyde; NS = non-significant; SOD = superoxide dismutase; LVEF = left ventricular ejection fraction.

[a]Shear rate = the rate of change of velocity at which one layer of fluid passes over an adjacent layer.

Additional file 2: Characteristics and results of the studies *continued*

Südhoff et al. [19]	Human	Malignant tumors<br>Treatment with 5-FU<br>(30 + 30 controls)	Brachial artery diameter (high resolution ultrasound)<br>Plasma concentrations of big endothelin	Contraction of the brachial artery in 15 of 30 patients<br>Vasocontraction more prevalent with infusional FU (66%) than bolus application (44%)<br>Reoccurrence of vasoconstriction in 86% of repeated administrations<br>Glyceroltrinitrate prevented vasocontraction<br>Trend towards increased big endothelin plasma levels	Arterial vasoconstriction leading to myocardial ischemia
Salepci et al. [18]	Human	Gastric or colon cancer<br>Treatment with 5-FU + leucvorin (31). Controls received non-5-FU regimens (28)	Brachial artery diameters<br>Angiotensin II and TnT assays	Decrease in brachial artery diameters<br>No change in angiotensin II and TnT levels	Arterial vasoconstriction leading to myocardial ischemia, not angiotensin II mediated
**Studies on RBCs**	**Type of study**	**Model**	**Parameters**	**Effects**	**Proposed mechanisms**
Spasojevic et al. [21]	In vitro	RBCs incubated in 5-FU	RBC morphology<br>Potassium efflux<br>Level of hemolysis<br>RBC membrane fluidity	Exposure-time/dose-dependent transformation of RBCs into echinocytic shape<br>Increased K+ efflux from RBCs into plasma<br>Decreased intracellular ATP levels<br>Membrane fluidity at concentrations of 10 mg/ml 5-FU	Alterations in RBCs leading to decreased oxygen transfer to the heart
Spasojevic et al. [22]	In vitro	Blood incubated in 5-FU	*pO2*<br>ATP and 2,3-BPG levels in RBCs	*pO2* in RBCs declined<br>2,3-BPG concentration in RBCs increased<br>Decreased ATP in RBCs	Alterations in RBCs leading to decreased oxygen transfer to the heart
Spasojevic et al. [23]	In vitro and human	Blood incubated in 5-FU<br>Patients treated with 5-FU and cisplatin (5)	*pO2*<br>ATP and 2,3-BPG levels in RBCs<br>31P NMR spectroscopy	Transformation of RBCs into echinocytic shape<br>Increased membrane fluidity<br>Decrease of *pO2*<br>Increased 2,3-BPG<br>Decreased ATP<br>Downfield shift in 31P-NMR spectra of blood samples (NS)	Alterations in RBCs leading to decreased oxygen transfer to the heart
Baerlocher et al. [20]	In vitro	Blood incubated in 5-FU	RBC morphology<br>Blood viscosity<br>Biophysical properties of RBCs and polymorph nuclear leucocytes	Dose-dependent, reversible echinocytic shape transformation of RBCs<br>Dose-dependent decrease in blood viscosity at low shear rates[a] and increase at high shear rates<br>No effect on plasma viscosity<br>Decreased RBC aggregation at high 5-FU concentrations (10 mg/ml)<br>Impaired transit of RBCs through 5 µm pores	Alterations in RBCs leading to decreased oxygen transfer to the heart

NOT = the results do not support the proposed mechanism
AOP = antioxidant potential; CAT = catalase; GSH-PX = glutathione peroxidase; MDA = malondialdehyde; NS = non-significant; SOD = superoxide dismutase; LVEF = left ventricular ejection fraction.
[a]Shear rate = the rate of change of velocity at which one layer of fluid passes over an adjacent layer.

Additional file 2: Characteristics and results of the studies *continued*

Cwikiel et al. [24]	Human	Male, squamous cell carcinomas Treatment with 5FU (11)	Blood viscosity<br>Plasma viscosity<br>Fibrinogen<br>Hemotocrit	Blood viscosity decreased<br>Plasma viscosity decreased<br>Fibrinogen values decreased<br>Correlation between blood viscosity and fibrinogen level before and during infusion	NOT increased blood viscosity leading to thrombosis
**Studies of substances in blood samples from human**	**Type of study**	**Model**	**Parameters**	**Effects**	**Proposed mechanisms**
Kuzel et al. [25]	Human	Patients with head and neck or gastrointestinal cancers treated with 5-FU +/- cisplatin (10)	Fibrinopeptide A (FpA)<br>Protein C activity (PCa)<br>Protein C (PCag)<br>Free protein S antigen	Rise in FpA activation during infusion<br>A reduction in PCa compared to PCag after infusion	-
Thyss et al. [28]	Human	Cancer patients<br>(8 with 5-FU induced cardiotoxicity, 8 5-FU-treated without cardiotoxicity, 8 treated with other chemotherapeutics and 30 healthy controls)	Endothelin-1	Higher plasma endothelin-1 levels in patients treated with 5-FU<br>Even higher plasma endothelin-1 levels in patients with 5-FU cardiotoxicity	Increased endothelin-1 leading to arterial vasoconstriction and myocardial ischemia
Jensen et al. [26, 27]	Human	Patients with colorectal cancer treated with 5-FU + oxaliplatin (106)	NT-proBNP<br>Lactic acid<br>Plasma von Willebrand factor (vWF)<br>Urine albumin-creatini ratio (UACR)<br>Coagulation facor II, VII and X<br>Fibrin D-dimer<br>LVEF	Increased NT-proBNP and plasma lactic acid<br>Higher NT-proBNP levels in patients with cardiotoxicity<br>Increased vWF, UACR and fibrin D-dimer<br>Decreased coagulation factor activity<br>No differences in vWF, UACR, coagulations factors and fibrin D-dimer between patients with or without cardiotoxicity<br>No changes in LVEF	NOT thrombosis.<br>NOT decreased cardiac pump function

NOT = the results do not support the proposed mechanism
AOP = antioxidant potential; CAT = catalase; GSH-PX = glutathione peroxidase; MDA = malondialdehyde; NS = non-significant; SOD = superoxide dismutase; LVEF = left ventricular ejection fraction.
[a]Shear rate = the rate of change of velocity at which one layer of fluid passes over an adjacent layer.
